# A collapse risk assessment method for subway foundation pit based on cloud model and improved Dempster–Shafer evidence theory

**DOI:** 10.1038/s41598-024-52643-x

**Published:** 2024-02-01

**Authors:** Bo Wu, Jiajia Zeng, Ruonan Zhu, Fan Yang, Cong Liu, Yundong Xie

**Affiliations:** 1 School of Civil and Architecture Engineering, East China University of Technology, Nanchang, 330013 Jiangxi China; 2 School of Water Resources and Environmental Engineering, East China University of Technology, Nanchang, 330013 Jiangxi China; 3 Jiangxi Geological Survey Research Institute, Nanchang, 330001 Jiangxi China; 4 China Railway Beijing Engineering Bureau Group Urban Rail Transit Engineering Co., Ltd, Hefei, 230000 Hui An China

**Keywords:** Computational science, Environmental impact, Civil engineering, Natural hazards

## Abstract

Collapse is a major engineering hazard in open-cut foundation pit construction, and risk assessment is crucial for considerably reducing engineering hazards. This study aims to address the ambiguity problem of qualitative index quantification and the failure of high-conflict evidence fusion in risk assessment. Thus, a fast-converging and high-reliability multi-source data fusion method based on the cloud model (CM) and improved Dempster–Shafer evidence theory is proposed. The method can achieve an accurate assessment of subway pit collapse risks. First, the CM is introduced to quantify the qualitative metrics. Then, a new correction parameter is defined for improving the conflicts among evidence bodies based on conflict degree, discrepancy degree and uncertainty, while a fine-tuning term is added to reduce the subjective effect of global focal element assignment. Finally, the risk assessment result is obtained according to the maximum affiliation principle. The method is successfully applied to Luochongwei Station, where the difference between the maximum value and the second largest value of the basic probability assignment is 0.624, and the global uncertainty degree is 0.087. Both values satisfy the decision evaluation condition; however, values of other methods only satisfy one or neither condition. In addition, the proposed method requires only four cycles to reach the steady state by fusing data of the same index, which has faster convergence compared with that of other methods. The proposed method has good universality and effectiveness in subway pit collapse risk assessment.

## Introduction

The subway is an important part of rail transportation; It attracts a high density of people and has a high capacity in urban areas, effectively relieving ground traffic congestion. However, unforeseen safety risks are associated with the complexity of a subway pit construction and the sensitivity of the surrounding environment. The open-cut method is widely used in subway pit excavation because of its low cost and high adaptability to the stratum. Owing to the numerous risk factors involved in pit construction, the pit is susceptible to collapses, which may result in economic losses and human casualties. Therefore, it is crucial to accurately assess the risks of pit construction collapse to ensure a safe subway pit construction^1–3^.

Common means of collecting data for a subway pit risk assessment exercise include survey and design, site inspection, and instrument-based monitoring. Owing to the complexity and sensitivity of the construction environment, a single data source may be considerably influenced by electromagnetic interference and human subjectivity. Additionally, a single data source cannot fully reflect the state of the construction site, and it may introduce fuzziness into the data. Therefore, multi-source information fusion methods are typically used to improve the reliability of the assessments^4,5^. For instance, the cloud model (CM) was used to quantify qualitative data in a study^6^. In another study, Yan used the CM to assess the tunneling risk of shield machines in soil–rock composite strata using monitoring data and relying on construction engineering experience^7^. Deng used the CM to characterize the uncertainty of evaluation factors and proposed a 3D urban geological suitability evaluation system^8^. These studies show that the CM can be used for data conversion in the information fusion assessment of subway pit risks.

However, owing to the different focus points and data collection levels of different information sources, harmonizing the data obtained is often difficult, and the data are typically prone to bias as well as high conflicts and mutual exclusion. Dempster–Shafer (D–S) evidence theory is widely used in the fields of data fusion, reliability assessment, and fault identification^9–11^. Shen proposed a risk evaluation method that combines fuzzy sets with D–S evidence theory to analyze the risks of deep-foundation pit construction under conditions of incomplete information^12^. Huang considered the arbitrary selection of fuzzy operators and improved the credibility of the shield tunnel risk assessment by using D–S evidence theory for the uncertainty inference of the confidence function^13^. Mokarram combined fuzzy hierarchical analysis and D–S evidence theory to predict karst suitability zones, and the results showed that the proposed method was superior to using only the fuzzy hierarchical analysis^14^. Further, Park proposed a data integration framework based on D–S evidence theory for predicting landslide sensitivity, and the method effectively integrates multiple datasets while achieving a higher prediction accuracy than that of the traditional logistic regression^15^. However, the method does not consider the high conflicting pieces of information; hence, other scholars have improved on the method^16–18^. As only a single conflict metric is considered, different application scenarios may face weak immunity, and the generalization ability of the method is insufficient to characterize the high degree of mutual exclusion accurately.

Therefore, this paper proposes a subway pit collapse risk assessment method based on the CM and improved D–S evidence theory. Three data sources, including survey and design, site inspection, and instrumented monitoring, are used, and video surveillance data are introduced. The CM is introduced to quantify qualitative metrics, and a new correction parameter is defined according to conflict, discrepancy, and uncertainty degrees. Additionally, evidence focal element assignment is considered to adjust the fusion rules to solve the high-conflict multi-data fusion failure problem, providing a new optimized idea and approach to subway pit risk fusion assessment.

## Methods

**Figure 1:**
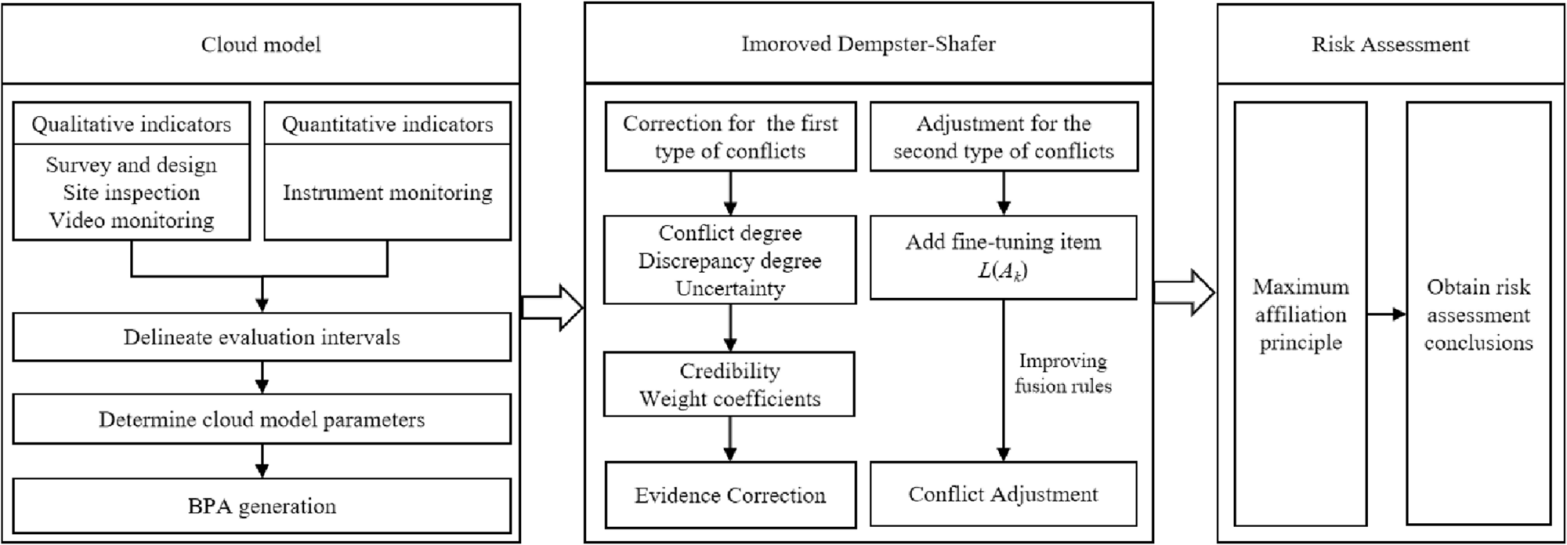
Flow chart of subway foundation pit collapse risk.

A multi-data fusion method based on the CM and improved D–S is proposed to improve the credibility and robustness of subway pit collapse risk assessments. The flow chart of the method is shown in Fig. 1. First, the risk assessment system and assessment set are constructed from an engineering case, and the numerical cloud characteristics and affiliation function of the assessment set are obtained using the CM. Conflict degree, discrepancy, and uncertainty are introduced as terms for the first type of conflict to obtain the credibility and weight coefficients of the different pieces of evidence. For the second type of conflict, fine-tuning terms are added to improve the fusion rules to adjust global conflicts. Finally, the overall risk assessment results of the foundation pit are derived according to the principle of maximum affiliation.

### BPA function construction based on CM

Let *U* be a quantitative theoretical domain expressed numerically and *C* be a qualitative concept in the theoretical domain *U*. If there exists a quantitative value *x* (x ∈ *U*), *x* is a random realization in *C*, and the affiliation *μ*(*x*) (*μ*(*x*) ∈ [0,1]) of *C* is a random number with a stable tendency, then the distribution of *μ*(*x*) in the theoretical domain *U* is simply called a cloud, and each value of *μ*(*x*) is called a cloud drop^19,20^. *Ex* is the expected value of the cloud drops’ distribution in *U*, and it describes the qualitative concept of cloud drops. *En* represents the qualitative concept of uncertainty, and it reflects the discrete degree of cloud drops. *He* is the uncertainty level of *En*, and it represents the degree of dispersion of *En*.

1 $$\left\{ {\begin{array}{*{20}l} {Ex = (a^{ - } + a^{ + } )/2} \\ {En = (a^{ + } - a^{ - } )/2.355} \\ {He = 0.01} \\ \end{array} } \right.$$

where *a*^−^ and *a*^+^ denote the lower and upper bounds of the assessment interval ([*a*^−^, *a*^+^]), respectively. As the attribute values given by the decision maker are stable, the degrees of dispersion are essentially the same. *He* is generally obtained empirically and is taken as 0.01 in this study.

Let the CM parameter of the risk level *A*_*k*_ obtained from the *s-*th indicator of the *i*-th data source be *Ex*_*isk*_, *En*_*isk,*_ and *He*_*isk*_, and the corresponding affiliation degree is *μ*_*is*_(*A*_*k*_). The affiliation degree is transformed into the basic probability assignment (BPA, *m*_*is*_(*A*_*k*_)), and the global uncertainty is *m*(Θ).

2 $$\left\{ \begin{gathered} \mu_{is} (A_{k} ) = \exp \left( { - \frac{{(x_{is} - Ex_{isk} )^{2} }}{{2(En^{\prime}_{isk} )^{2} }}} \right) \hfill \\ En^{\prime}_{isk} = En_{isk} + He_{isk} *Rand(0,1) \hfill \\ m_{is} (\Theta ) = 1 - \max (\mu_{is} (A_{k} )) \hfill \\ m_{is} (A_{k} ) = (1 - m_{is} (\Theta ))\frac{{\mu_{is} (A_{k} )}}{{\sum\nolimits_{k = 1}^{p} {(\mu_{is} (A_{k} )|i,s)} }} \hfill \\ \end{gathered} \right.$$

where $En^{\prime}_{isk}$ is a normal random number that satisfies an expectation of *En*_*isk*_ and a standard deviation of *He*_*isk*_. *i* denotes the number of data sources. *s* denotes the number of indicators for the data source. *k* denotes the number of risk levels, and the value is taken as 4 in this study.

### Improved evidence conflict method

Evidence conflicts can be categorized into two types according to the evidence conflict fusion process. The first type is a conflict between evidence bodies, which results from the bodies of evidence themselves. The second type is the flaw of the fusion rule^21,22^. For the risk assessment in this study, two types of evidence bodies are considered: different indicator evidence bodies of the same data source (Internal Evidence Body, IEB) and different data-source evidence bodies (External Evidence Body, EEB). In this study, IBE is used to illustrate the fusion process.

For the first type of conflicts, multiple indicators are considered, including conflict degree (*α*), variance degree (*β*), and uncertainty degree (*γ*). Conflict degree (*α*) is expressed as a conflict factor, which indicates the overall conflict between evidence bodies. Let $\alpha_{i}^{st}$ be the conflict degree between the *s*-th and *t*-th indicator evidence bodies of the *i*-th data source.

3 $$\alpha_{i}^{st} = \sum\limits_{s \ne t} {m_{i}^{s} \cdot m_{i}^{t} }$$

where $m_{i}^{s}$ and $m_{i}^{t}$ denote the mass function of the *s*-th and *t*-th indicator of the *i*-th data source, respectively.

The variance degree (*β*) is expressed in terms of Euclidean distance, which describes the similarity between the pieces of evidence. Let $\beta_{i}^{st}$ be the difference degree between the *s*-th and *t*-th indicators of the *i*-th data source.

4 $$\beta_{i}^{st} = \sqrt {\sum\limits_{s \ne t} {(m_{i}^{s} - m_{i}^{t} )^{2} } }$$

To maintain the same monotonicity among conflict degree *α*, variance degree *β*, and uncertainty degree *γ*, the focusing degree *θ* is introduced to represent the uncertainty degree *γ*. The focusing degree *θ* indicates the uncertainty of a single evidence body itself. The greater the focusing degree *θ*, the smaller the uncertainty degree *γ*. Let $\theta_{i}^{s}$ denote the focusing degree of the *s*-th indicator of the *i*-th data source, and its corresponding uncertainty degree is $\gamma_{i}^{s}$.

5 $$\gamma_{i}^{s} = 1 - \theta_{i}^{s} = 1 - \sqrt {\frac{1}{\left| \Theta \right|}\sum {\left( {m_{i}^{s} - \frac{1}{\left| \Theta \right|}} \right)^{2} } }$$

where |Θ| is the cardinality of the subset Θ.

**Figure 2:**
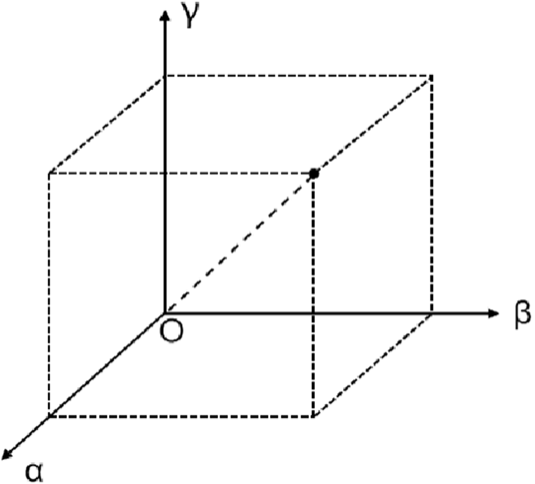
Improved evidence correction parameter 3D space vector.

The conflict degree (*α*), variance degree (*β*), and uncertainty degree (*γ*) have the same monotonicity. The larger the values of the three indicators above, the greater the value of the evidence conflict. The stereoscopic space is introduced to optimize the D–S evidence theory by projecting α, β, and γ onto the x-axis, y-axis, and z-axis, respectively, as shown in Fig. 2. The dynamic weight coefficient method is used to determine the weight coefficients of different pieces of evidence. The spatial distance from (α, β, γ) to (0, 0, 0) is introduced in this study. A new conflict parameter ($dis_{i}^{s}$) can be obtained after monotonicity consistency processing. Since the new parameter ($dis_{i}^{s}$) varies with evidence, it is normalized to obtain the weight coefficients (*w*^*s*^).

6 $$(m_{i}^{s} (A_{k} ))^{\prime} = w^{s} m_{i}^{s} (A_{k} )$$

7 $$\left\{ \begin{gathered} dis_{i}^{s} = 1 - \sqrt {\frac{{(\alpha_{i}^{s} )^{2} + (\beta_{i}^{s} )^{2} + (\gamma_{i}^{s} )^{2} }}{3}} \hfill \\ w^{s} = \frac{{dis_{i}^{s} }}{{\sum\nolimits_{s = 1}^{q} {dis_{i}^{s} } }} \hfill \\ \end{gathered} \right.$$

where ($m_{i}^{s}$(*A*_*k*_))′ denotes the BPA of the *s*-th indicator evidence body of the *i*-th data source to the *k*-th target *A*_*k*_ after correction, and 0 ≤ ($m_{i}^{s}$(*A*_*k*_))′ ≤ 1, 0 ≤ ($m_{i}^{s}$(Θ))′ ≤ 1, *s* = 1, 2,…, *q*, *k* = 1, 2,…, *p.*

The above correction method solves the weight proportion of the BPA and eliminates the differences among evidence bodies. However, the method fails to solve the global focal element assignment among evidence bodies, making the allocation of global focal elements more subjective. Therefore, the *L*(*A*_*k*_) is added to decompose the global conflicts to local conflicts, which can eliminate the difference among pieces of evidence. Let (*m*(*A*_*k*_))″ denote the mass function of the *s*-th and *t*-th indicator evidence of the *i*-th data source fused against the target *A*_*k*_. *L*(*A*_*k*_) is the corresponding fine-tuning term.

8 $$\left\{ \begin{gathered} L(A_{k} ) = \sum\nolimits_{{C \cap D = A_{k} }} {(m_{i}^{s} (C))^{\prime} \cdot (m_{i}^{t} (D))^{\prime}} \hfill \\ (m(A_{k} ))^{*} = (m_{i}^{s} (C))^{\prime} + (m_{i}^{t} (D))^{\prime} + L(A_{k} ) \hfill \\ (m(A_{k} ))^{\prime\prime} = \frac{{(m_{i}^{s} (A_{k} ))^{*} }}{{\sum\nolimits_{{A_{k} \subseteq \Theta }} {(m_{i}^{s} (A_{k} ))^{*} } }} \hfill \\ \end{gathered} \right.$$

where *C* and *D* denote subevents of event *A*_*k*_.

After fusion, the mass matrix is [($m_{i}^{s}$(*A*_*1*_))″*,* ($m_{i}^{s}$(*A*_*2*_))″, *…*, ($m_{i}^{s}$(*A*_*k*_))″, *…*, ($m_{i}^{s}$(*A*_*p*_))″*,* ($m_{i}^{s}$(Θ))″]. Additionally, ($m_{i}^{s}$(Θ))″ ≤ 0.1, max(($m_{i}^{s}$(*A*_*k*_))″)–max(($m_{i}^{s}$(*A*_*p*_))″) ≥ 0.2, *k ≠ p.*

## Results

### Engineering background

**Figure 3:**
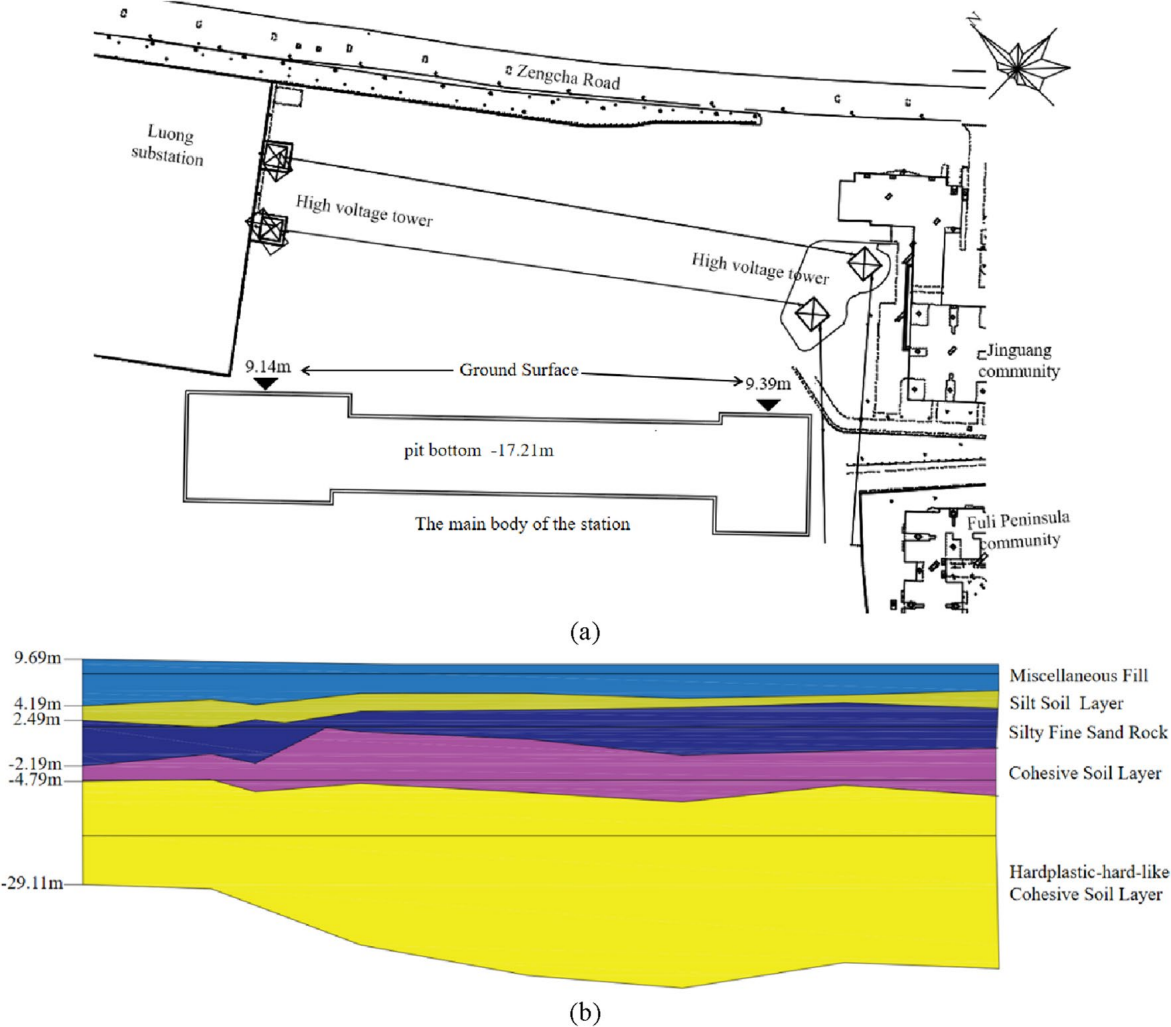
(**a**) Station plan. (**b**) Section view of station.

This study was based on the Luochongwei Station of Guangzhou Metro Line 13. The BPA of different data sources was constructed using the CM. To verify the effectiveness of the proposed method, the improved D–S theory was applied to assess the risk of construction safety. The station is an underground three-level side station with a total length of 220 m, a total construction area of 24,570 square meters, and a standard section width of 34 m. Open excavation is performed in the project at an excavation depth of approximately 24 m. The main enclosure structure adopts the system of a 1-m underground diaphragm wall and internal support. The station has a complex surrounding environment, and the station plan is shown in Fig. 3a. Additionally, the station has poor geological conditions, a nearby fracture zone, and abundant groundwater. The section view of the station is shown in Fig. 3b.

### Survey and design index system

The survey and design index is semi-qualitative. Original data were obtained through expert scoring for a semi-qualitative index. To establish the risk assessment model, the assessment score was set to 10 points. The higher the score, the greater the risk. The assessment level was divided into four risk levels: I, II, III, and IV. The risk increases sequentially from I to IV^23^. The risk index grading criteria based on the survey and design are presented in Table 1.

**Table 1:** Risk indicator grading criteria and acceptance guidelines based on survey and design.

Risk level	Level I	Level II	Level III	Level IV
Score	[0, 2.5)	[2.5, 5)	[5, 7.5)	[7.5, 10]
Acceptance guidelines	Acceptable	No expectations	Hard to accept	Unacceptable
Processing measures	Less risky, can maintain status quo	Higher risk, need to pay close attention	High risk, need to identify causes and control risks	The risk is very high, need to start the emergency plan, take immediate measures to reduce the risk

The foundation excavation has an impact on the surrounding environment, which can be determined by the foundation excavation parameters, the location and state of the surrounding buildings, and the hydrogeological conditions. Such influences are determined when the engineering survey and design are completed, which are also closely related to the risk of pit construction^24^. Risk indicators based on survey and design can be divided into three categories^25^, namely, the inherent properties of the pit (E1), which can reflect the disaster losses; hydrogeological indicators (E2), which can reflect the possibility of damage to the pit; and the conditions of the surrounding structures (E3), which can reflect the possibility of damage to the structures. The statistical results are presented in Table 2.

**Table 2:** Risk indicators and ranking table based on survey and design data (E).

Indicators		Risk level			
		Level I	Level II	Level III	Level IV
Inherent properties of pit (E1)	Excavation depth (E11)	H < 7 m	7 ≤ H < 13 m	13 m ≤ H < 17 m	H ≥ 17 m
	Excavation area (E12)	S < 10 km^2^	10 km^2^ ≤ S < 20 km^2^	20 km^2^ ≤ S < 50 km^2^	S ≥ 50 km^2^
Hydrogeology (E2)	Groundwater type and burial conditions (E21)	No effect	Pressurized water; clear burial conditions	High pressurized head; more complex burial	High pressurized head; complex burial
	Soil quality and stratification (E22)	High soil strength; simple layering	Average soil strength; clearer layering	Lower soil strength; more complex layering	Very low soil strength; complex layering conditions
	Corrosion of materials by soil and water (E23)	Micro corrosive	Weakly corrosive	Medium corrosive	Strong corrosive
Surrounding buildings and structures (E3)	Ratio of distance to pit depth (E31)	K > 2	1 < K ≤ 2	0.5 < K ≤ 1	K ≤ 0.5
	Adjacency type (E32)	None	Shorter	Longer	Horizontal projection with crossover
	Initial deformation (E33)	Minor	Moderate	Large	Extremely large
	Initial leakage (E34)	No seepage	Small leakage	Multiple water seepage	Multiple drips and thin streams

### Instrument monitoring index system

The instrument monitoring index is quantitative. Original data were obtained from on-site measurements for the quantitative index. The risk assessment indicators selected were surface settlement (F1), groundwater level (F2), horizontal displacement of wall top (F3), and vertical displacement of column (F4)^23,26,27^. These four indexes depend on both cumulative values and change rates, as shown in Table 3. As the results of the risk assessment and the actual monitoring values had the same trend, the early-warning value was used as a baseline, and the index was converted to a dimensionless quantity by *K* to harmonize risk indicators. *K* is the ratio of the actual monitoring value to the early-warning value. The risk indicators were divided into four levels using 60%, 80%, and 100% of the early-warning value, as shown in Table 4.

**Table 3:** Foundation pit monitoring control value standard.

Monitoring Items	Early-warning value	
	Cumulative value	Rate of change
Surface settlement	24 mm	2.5 mm/d
Groundwater level	1.6 m	0.5 m/d
Horizontal displacement of wall top	24 mm	2 mm/d
Vertical displacement of column	12 mm	1.4 mm/d

**Table 4:** Risk indicators and ranking table based on instrument monitoring data (F).

Indicators		Risk level			
		Level I	Level II	Level III	Level IV
Surface settlement (F1)	Cumulative value (F11)	0 < K < 0.6	0.6 < K < 0.8	0.8 < K < 1	1 < K < 1.2
	Rate of change (F12)				
Groundwater level (F2)	Cumulative value (F21)				
	Rate of change (F22)				
Horizontal displacement of wall top (F3)	Cumulative value (F31)				
	Rate of change (F32)				
Vertical displacement of column (F4)	Cumulative value (F41)				
	Rate of change (F42)				

### Site inspection index system

The site inspection index is qualitative. Original data were obtained from expert scoring for the qualitative index; the criteria for expert scoring are presented in Table 1. The instrument monitoring data cannot fully reflect the construction risks, such as cracks and water seepage in the foundation pit. The site inspection indicators are an extension of the instrument monitoring indicators, which focus on the items that are difficult to quantify. The risk assessment indicators considered were construction work conditions (G1), support structure (G2), surroundings (G3), and monitoring facilities (G4)^28–30^. The risk-level classification criteria for the site inspection indicators are consistent with the survey and design indicators, which are also classified into four levels^23^, as shown in Table 5.

**Table 5:** Risk indicators and level classification table based on site inspection data.

Indicators		Risk level			
		Level I	Level II	Level III	Level IV
Construction work conditions (G1)	Consistency of soil with Survey Report (G11)	Exactly the same	Basically the same	Inconsistent	Completely inconsistent
	Geotechnical stability (G12)				
	Precipitation, recharge facilities status (G13)	Qualified	Basic pass	Unqualified	Completely unqualified
	Piling around the pit (G14)				
	Interception and drainage effect (G15)				
Support structure (G2)	Support structure (G21)	Qualified	Basic pass	Unqualified	Completely unqualified
	Support pile (wall) cracks (G22)				
	Column, support tilt deformation (G23)	Minor	Moderate	Large	Extremely large
	Quicksand, pit bottom rises (G24)				
	Leakage of water stop curtain (G25)	Little water, no mud and sand	Little water, little mud and sand	Little water, large mud and sand	Large water, large mud and sand
	Timeliness of support erection (G26)	On time	Mostly timely	Untimely	Very untimely
Surroundings (G3)	Surrounding buildings (G31)	Qualified	Basic pass	Unqualified	Completely unqualified
	Surrounding river banks (G32)				
	Surrounding road (G33)				
	Leakage of underground pipelines (G34)				
	Surrounding activities (G35)				
	Water seepage of underground structures (G36)	No seepage	Small leakage	Multiple water seepage	Multiple drips, thin streams
Monitoring facilities (G4)	Complete status of monitoring points and elements (G41)	Complete	Basically complete	Incomplete	Very Incomplete
	Monitoring frequency, timeliness of warning (G42)	On time	Mostly timely	Untimely	Very untimely
	Monitoring data errors (G43)	No error	Smaller	Larger	Very large

### Video surveillance index system

The video surveillance index is qualitative. The original data were obtained from expert scoring for the qualitative index; the criteria for expert scoring are presented in Table 1. Video surveillance was introduced to assess the management risk of construction sites, which are divided into remote monitoring, medium-range monitoring, and near-range monitoring for different construction scenarios^31,32^. The large-scenario risk monitoring (H1) is characterized by a high number of risk target subjects, a wide spatial scope of risk distribution, and a scattered distribution of risk sources. The medium-scenario risk monitoring is characterized by a clear and limited number of risk target subjects, a more fixed risk distribution area, and a more concentrated distribution of risk sources. The small-scenario risk monitoring is characterized by uniquely identified risk target subjects, uniquely identified risk distribution locations, and risk sources concentrated in very small spatial areas. The unsafe factors of the pit contain five aspects: human, machine, material, method, and environment^33^. The risk indicators and level classification of different scale scenes were obtained according to the characteristics of monitoring scenes, as shown in Table 6.

**Table 6:** Risk indicators and level classification table based on video surveillance data (H).

Indicators		Risk level			
		Level I	Level II	Level III	Level IV
Large scenario risk (H1)	Worker’s safety equipment wear (H11)	Qualified	Basic pass	Unqualified	Completely unqualified
	Worker’s irregularities and violations (H12)				
	Lighting in the work area (H13)				
	Hygiene in the work area (H14)				
	Loading and unloading of transport machinery (H15)				
Medium scenario risk (H2)	Raw material storage (H21)				
	Hazardous materials storage (H22)				
	Construction waste deposition (H23)				
	Protective facilities in hazardous areas (H24)				
	Warning signs set in hazardous areas (H25)				
Small scenario risk (H3)	Construction process specification (H31)				
	Large machinery and equipment parking, operating norms (H32)				

### BPA generation for different data sources

Survey and design data were collected before pit excavation, which was finalized after the design was completed. Instrument monitoring, site inspection, and video surveillance were performed after pit excavation, and the data were collected once daily. The data from one day after pit excavation are used as an example to illustrate the process of the proposed method.

**Figure 4:**
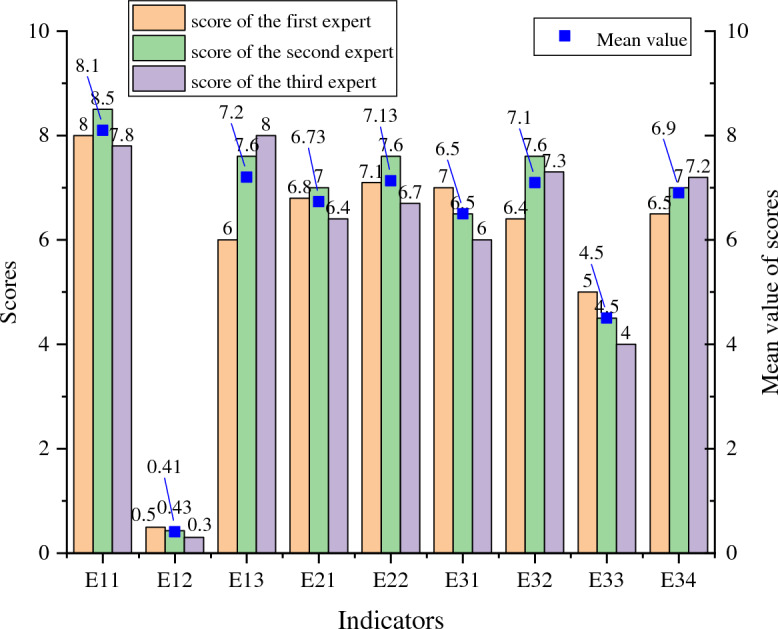
Cloud model. (**a**) Survey and design, site inspection, video monitoring. (**b**) Instrument monitoring.

**Figure 5:**
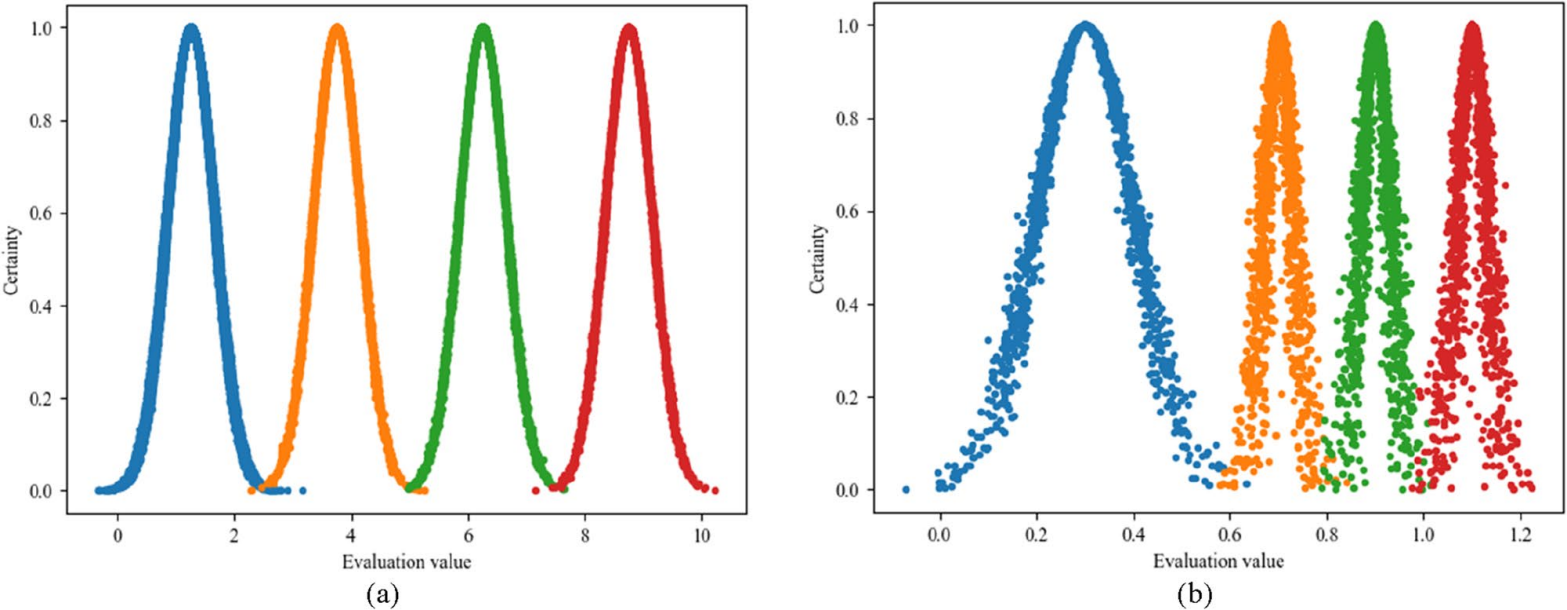
Expert scoring results of the survey and design indicators.

1. For the survey and design, site inspection, and video surveillance, the main processes are as follows. First, the experts score the indicators. The scoring results from three experts are averaged to obtain the score corresponding to each indicator. Next, according to Eq. (1), the set of survey and design rubrics is converted to digital cloud features. Then, according to Eq. (2), the scores are converted to an affiliation. Finally, according to Eq. (2), the affiliation is converted to BPA.
  Taking the survey and design as an example, we note that the survey and design index system contains three secondary indicators: E1, E2, and E3. The corresponding tertiary indicators are E11, E12, E13, E21, E22, E31, E32, E33, and E34. The set of risk identification levels are I, II, III, IV, and Θ, where Θ denotes the uncertainty of the global, which indicates the unknown levels. When E11 is taken as an example, first, the average of the scores assigned by the three experts is 8.1, as shown in Fig. 4. Next, the standard numerical cloud characteristics (Table 7) and the cloud diagram (Fig. 5) can be obtained. As can be seen in Fig. 5, no overlap occurs between the sets of rubrics, which can be used for the conversion of data for each risk source. The cloud digital characteristics of the survey and design are $$\left[ \begin{gathered} \begin{array}{*{20}c} {1.25} & {1.062} & {0.01} \\ \end{array} \hfill \\ \begin{array}{*{20}c} {3.75} & {1.062} & {0.01} \\ \end{array} \hfill \\ \begin{array}{*{20}c} {6.25} & {1.062} & {0.01} \\ \end{array} \hfill \\ \begin{array}{*{20}c} {8.75} & {1.062} & {0.01} \\ \end{array} \hfill \\ \end{gathered} \right]$$. Then, *En*′ follows NORMINV(*p*, 1.062, 0.01) and *p* is rand(0,1), so *En*′ is [1.057, 1.057, 1.057, 1.057]. [*u*_11_(*A*_1_), *u*_11_(*A*_2_), *u*_11_(*A*_3_), *u*_11_(*A*_4_)] are [0, 0, 0.217, 0.828]. Finally, the affiliation is converted to BPA. BPA(E11) is [0, 0, 0.17, 0.657, 0.173]. The BPA of other indicators can also be calculated using the above method, as shown in Table 8.

**Table 7:** Cloud digital characteristics of different data sources.

Data source	Evaluation interval	Cloud digital characteristics (*Ex*, *En*, *He*)
Survey and design (E) Site inspection (F) Video monitoring (H)	[0.0, 2.5]	(1.25, 1.062, 0.01)
	[2.5, 5.0]	(3.75, 1.062, 0.01)
	[5.0, 7.5]	(6.25, 1.062, 0.01)
	[7.5, 10]	(8.75, 1.062, 0.01)
Instrument monitoring (G)	[0.0, 0.6]	(0.30, 0.255, 0.01)
	[0.6, 0.8]	(0.70, 0.085, 0.01)
	[0.8, 1.0]	(0.90, 0.085, 0.01)
	[1.0, 1.2]	(1.10, 0.085, 0.01)

**Table 8:** BPA of survey and design.

Indicators	m(I)	m(II)	m(III)	m(IV)	m(Θ)
E11	0.000	0.000	0.170	0.657	0.173
E12	0.725	0.007	0.000	0.000	0.268
E13	0.000	0.003	0.440	0.225	0.332
E21	0.000	0.016	0.748	0.137	0.098
E22	0.000	0.004	0.487	0.215	0.293
E31	0.000	0.028	0.857	0.087	0.028
E32	0.000	0.006	0.511	0.216	0.267
E33	0.007	0.581	0.191	0.000	0.221
E34	0.000	0.009	0.649	0.170	0.172
  The cloud model solves the problem of transforming uncertainty between qualitative concepts and quantitative values. The comment set of risk sources is converted into cloud digital features, and, finally, the expert scoring results (survey and design, site inspection and video surveillance) are converted into BPA. The BPA value obtained is used to fuse the information from multiple sources.
2. For instrument monitoring, the main processes are as follows. First, according to Eq. (1), the set of instrument monitoring rubrics is converted to cloud digital features. Next, according to Table (4), the measured values are converted to *K*. Then, according to Eq. (2), *K* is converted to an affiliation. Finally, according to Eq. (2), the affiliation is converted to BPA.

**Figure 6:**
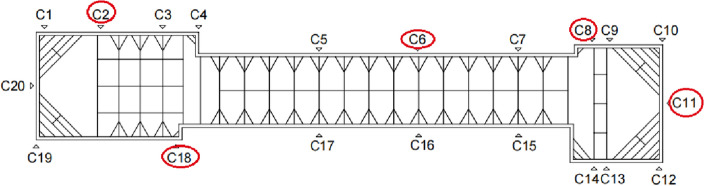
Monitoring point arrangement of Luochongwei station.

**Figure 7:**
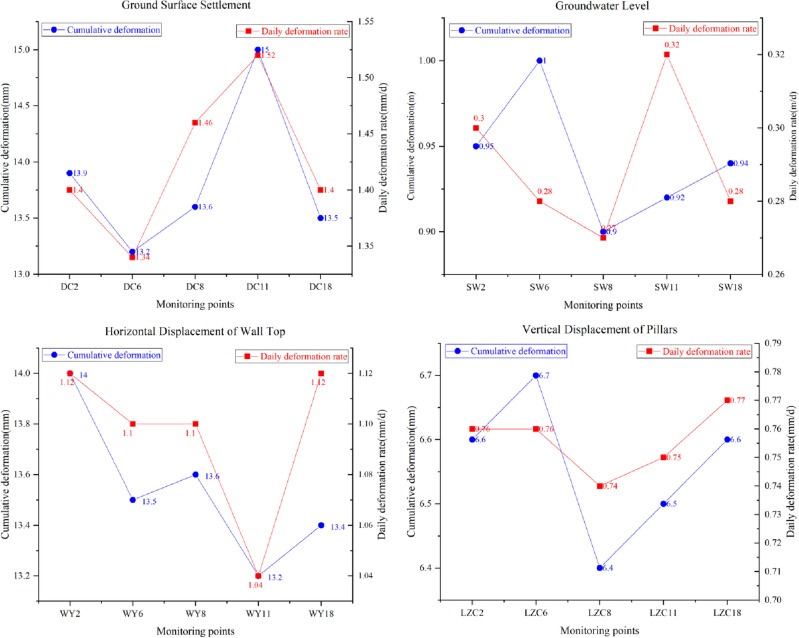
Instrument monitoring data.

The instrument monitoring index system contains four secondary indicators: F1, F2, F3, and F4. The corresponding tertiary indicators are F11, F12, F21, F22, F31, F32, F41, and F42. Each tertiary indicator corresponds to five monitoring points, and one moment of data is collected at each monitoring point. The set of risk identification levels are I, II, III, IV, and Θ. The Luochongwei Station contains a total of 20 monitoring sites. Figure 6 shows the monitoring points distribution, which has the same distribution principle for the monitoring points with a similar deformation. Therefore, five representative monitoring points (C2, C6, C8, C11, and C18) are selected for analysis in this paper. The corresponding measured data are shown in Fig. 7.

Taking monitoring point DC2 as an example, the cumulative value of the surface settlement is 13.9 mm. First, the standard cloud numerical characteristics (Table 7) and the cloud diagram (Fig. 5) can be obtained. As can be seen in Fig. 5, no overlap occurs between the sets of rubrics, which can be used for the data conversion of each risk source. The digital cloud characteristics of the instrument monitoring is $$\left[ \begin{gathered} \begin{array}{*{20}c} {0.3} & {0.085} & {0.01} \\ \end{array} \hfill \\ \begin{array}{*{20}c} {0.7} & {0.085} & {0.01} \\ \end{array} \hfill \\ \begin{array}{*{20}c} {0.9} & {0.085} & {0.01} \\ \end{array} \hfill \\ \begin{array}{*{20}c} {1.1} & {0.085} & {0.01} \\ \end{array} \hfill \\ \end{gathered} \right]$$. Next, the measured data of 13.9 mm is converted into *K* = 0.579. Then, *En*′ follows NORMINV(*p*, *En*, 0.01), *p* is rand(0,1), and *En* is [0.255, 0.085], so *En*′ is [0.258, 0.088, 0.088, 0.088]. [*u*_21_(*A*_1_), *u*_21_(*A*_2_), *u*_21_(*A*_3_), *u*_21_(*A*_4_)] is [0.558, 0.392, 0.001, 0]. Finally, the affiliation is converted to BPA. BPA(F21) is [0.323, 0.248, 0.17, 0.002, 0.427]. The BPA of other indicators can also be calculated using the above method, as shown in Table 9.

**Table 9:** BPA of surface settlement.

Indicator	Monitoring points	Indicators	m(I)	m(II)	m(III)	m(IV)	m(Θ)
Surface settlement (F1)	DC2	Cumulative value	0.323	0.248	0.002	0.000	0.427
		Rate of change	0.427	0.151	0.000	0.000	0.422
	DC6	Cumulative value	0.456	0.167	0.000	0.000	0.377
		Rate of change	0.530	0.117	0.000	0.000	0.353
	DC8	Cumulative value	0.396	0.163	0.000	0.000	0.441
		Rate of change	0.319	0.193	0.000	0.000	0.488
	DC11	Cumulative value	0.254	0.378	0.001	0.000	0.367
		Rate of change	0.265	0.315	0.002	0.000	0.417
	DC18	Cumulative value	0.402	0.183	0.000	0.000	0.415
		Rate of change	0.399	0.218	0.001	0.000	0.382

Similarly, the cloud model converts the uncertainty between the qualitative concept (instrument monitoring value) and quantitative value. The difference is that for the survey and design, site inspection, and video monitoring, the value of *K* is obtained through expert scoring. In contrast, instrument monitoring does not require expert scoring, but measured values are used directly to obtain the value of *K*, which is ultimately converted into a basic probability assignment. The basic probability assignments obtained from instrument monitoring, as well as those from survey design, site inspection, and video surveillance, are used to fuse the information from multiple sources.

### Multi-source data fusion

The accuracy of the collapse risk assessment from a single source of information is low and cannot provide accurate guidance for on-site construction because of reasons such as data errors that do not fully reflect the actual situation at the site. Therefore, this paper proposes a feature-based information fusion model, which can learn new-evidence correction parameters and reduce the impact of global uncertainty. Survey and design, site inspection, instrument monitoring, and video surveillance are used as the information sources for the collapse risk assessment, and the probability distribution of the corresponding collapse risk level is obtained from different information sources. To solve the problem of a large bias in the evaluation results of single information sources, the improved D–S evidence theory is used to fuse the data from multiple information sources. The method combines the above three single information source features and obtains the overall collapse risk results by fusing the judgment of each evidence. Survey and design indicators E11 and E12 are selected to illustrate the fusion process within the same risk source to obtain survey and design fusion results. Similarly, on-site inspection fusion results, instrument monitoring fusion results, and video surveillance fusion results can be obtained. The survey design (E) and on-site inspection (G) are selected to illustrate the fusion process of different risk sources so as to obtain the final risk assessment results. The fusion results of the single information source and the improved D–S theory of this paper are compared, as shown in Table 10. The following conclusions can be obtained.

**Table 10:** Final fusion results.

Data sources	m(I)	m(II)	m(III)	m(IV)	m(Θ)	Fusion level	Actual level
Survey and design (E)	0.000	0.200	0.710	0.000	0.090	III	II
Site inspection (G)	0.011	0.930	0.019	0.000	0.040	II	
Video monitoring (H)	0.000	0.880	0.100	0.000	0.020	II	
Instrument monitoring (F)	0.720	0.100	0.080	0.000	0.100	I	
Fusion results	0.160	0.750	0.000	0.007	0.083	II

1. The multi-source information fusion model has a good fault tolerance and can improve the error evaluation results by correcting the correction parameters of the correct evaluation results. The body of evidence of indicators from each data source is first fused sequentially to obtain the BPA for the four data sources, which are then fused sequentially to obtain the final fusion result. Nine tertiary indicators are used for the survey and design and need to be fused eight times. The results are shown in Fig. 8a. Site inspection needs to be fused 19 times, and the results are shown in Fig. 8b. Video surveillance needs to be fused 11 times, and the results are shown in Fig. 8c. The instrument monitoring contains eight tertiary indicators with five monitoring points for each indicator, creating a total of 40 sets of data that need to be fused 39 times. The results are shown in Fig. 8d.

**Figure 8:**
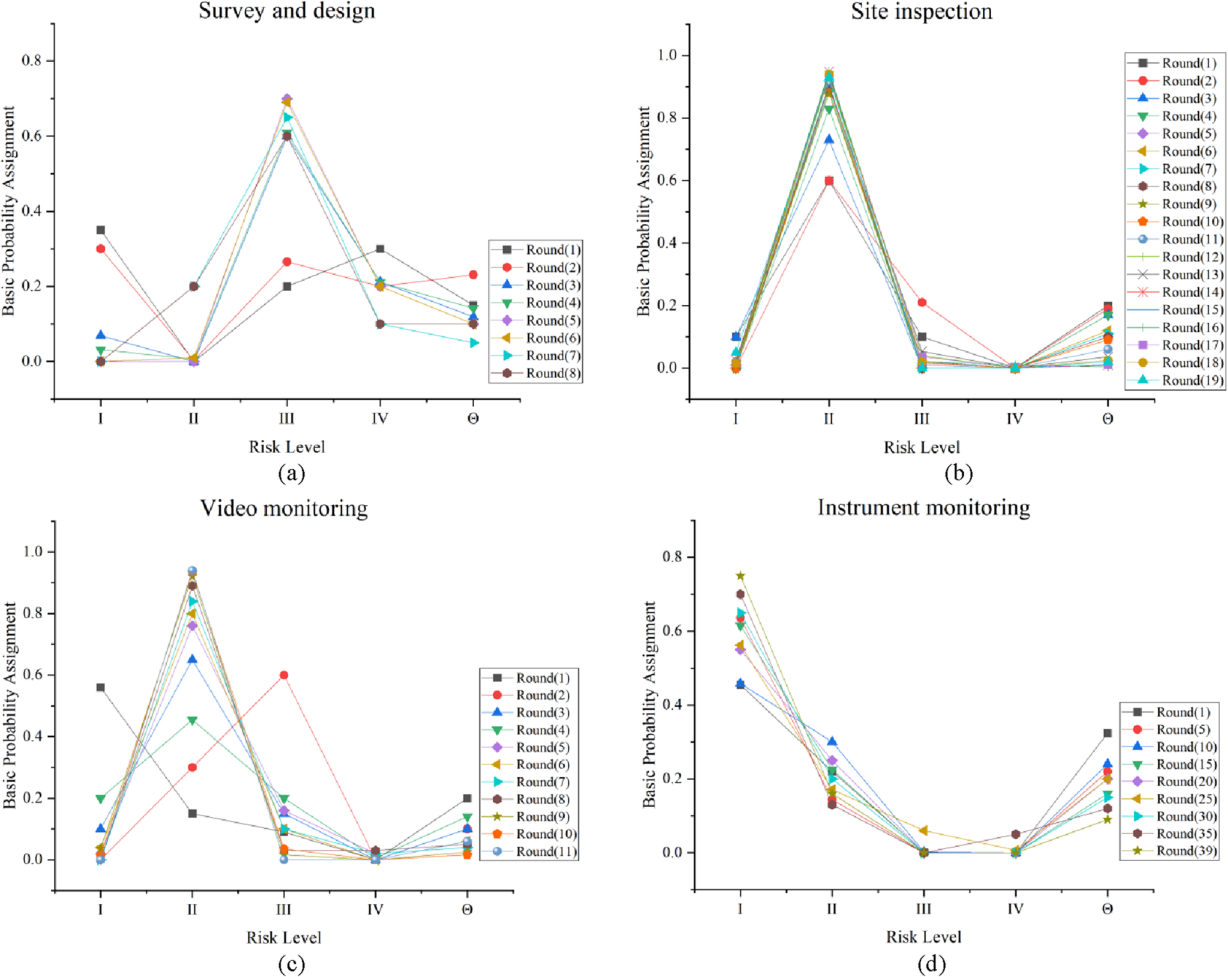
Fusion results. (**a**) Survey and design. (**b**) Site inspection. (**c**) Video monitoring. (**d**) Instrument monitoring.
  The fusion process is also described by survey and design indicators. BPA(E11) is [0, 0, 0.17, 0.657, 0.173], and BPA(E12) is [0.725, 0.007, 0, 0, 0.268]. According to Eqs. (3–7), the conflict degree (α) is 0.605, the variance degree (β) is 0.998, the uncertainty degree (γ) is [0.725, 0.68], and the corresponding weighting coefficients are 0.485 and 0.515. The corrected fused BPA is [0.412, 0, 0.088, 0.27, 0.23] according to Eq. (8). Then, the fusion result is fused with E13, and the final fused BPA of the survey and design indicators is [0, 0.2, 0.71, 0, 0.09]. *m*(Θ) is 0.09, which is less than or equal to 0.1, and *max*(*m*(*A*_*k*_))–*max*(*m*(*A*_*p*_), *k* ≠ *p*) is 0.51, which is greater than or equal to 0.2. Therefore, the fusion results satisfy the decision assessment conditions, and the risk assessment level based on the survey and design data is level II. Similarly, the final fused BPA of the site inspection is [0.011, 0.93, 0.019, 0, 0.04], and its risk assessment level is II. The final fused BPA of the video surveillance is [0, 0.88, 0.1, 0, 0.02], and its risk assessment level is II. The final fused BPA of the instrument monitoring is [0.72, 0.1, 0.08, 0, 0.1], and its risk assessment level is I. The final risk assessment result is obtained by fusing the four pieces of evidence, as shown in Table 10.
  The entire fusion process is explained as follows. The survey and design (E) and site inspection (G) are first integrated. BPA(E) is [0, 0.2, 0.71, 0, 0.09], and BPA(G) is [0.011, 0.93, 0.019, 0, 0.04]. According to Eqs. (3–7), α is 0.58, β is 0.951, γ is [0.745, 0.588], and the corresponding weighting coefficients are 0.453 and 0.547. According to Eq. (8), the corrected fused BPA (BPA(E ⊕ G)) is [0.01, 0.62, 0.3, 0.01, 0.06]. This fusion result is then fused with the video surveillance evidence body (H). BPA(E ⊕ G) is [0.01, 0.62, 0.3, 0.01, 0.06], and BPA(H) is [0, 0.88, 0.1, 0, 0.02]. α is 0.346, β is 0.331, γ is [0.73, 0.614], and the corresponding weighting coefficients are 0.476 and 0.524. Hence, the corrected fused BPA (BPA(E ⊕ G ⊕ H)) is [0.1, 0.83, 0.06, 0, 0.01]. Finally, the above fusion result is fused with the instrument monitoring evidence body (F). BPA(E ⊕ G ⊕ H) is [0.1, 0.83, 0.06, 0, 0.01], and BPA(F) is [0.72, 0.1, 0.08, 0, 0.1]. α is 0.731, β is 0.962, γ is [0.641, 0.701], and the corresponding weighting coefficients are 0.521 and 0.479. Hence, the corrected fused BPA (BPA(E ⊕ G ⊕ H ⊕ F)) is [0.16, 0.75, 0, 0.007, 0.083]. *m*(Θ) is 0.083, which is less than or equal to 0.1. *max*(*m*(*A*_*k*_))–*max*(*m*(*A*_*p*_), *k* ≠ *p*) is 0.59, which is greater than or equal to 0.2. Therefore, the final fusion results satisfy the decision assessment conditions, and the final risk level is II. The values of the elements in the mass function are clearly distinguished, and no case of the values of the elements being similar in size exists; moreover, and the risk assessment results are accurate, indicating the effectiveness and stability of the proposed method.
2. When assessing collapse risk, the results are often biased because of the uncertainty of the data from a single source of information. The proposed method synthesizes information from different sources (including conflicting information) to provide a comprehensive view of construction, thereby reducing the data uncertainty and improving the assessment accuracy. Therefore, the results of multi-source information fusion assessments tend to have a higher accuracy than that of single-source information risk assessment methods.

The single-source information model cannot provide accurate decision-making opinions for on-site construction. This is because a single source of information does not adequately consider the risk factors of pit collapse; moreover, it contains errors and uncertainties, which ultimately leads to a slight deviation in the assessment results from the actual situation. The proposed method fully utilizes the available information and includes conflict information. The proposed model considers the four risk source data of survey and design, site inspection, instrument monitoring, and video surveillance; thus, the results of the evaluation model are closer to the actual situation while having an improved accuracy. These results prove the effectiveness and feasibility of applying the evaluation method in an actual construction process.

## Discussion

To compare different multi-source information fusion methods, the proposed method and other previous methods are used to evaluate the collapse risk of the same indicators. Site inspection data were selected for risk assessment, and the model was evaluated from two perspectives: model validity and convergence, respectively. The analysis and conclusions are as follows.

1. The fusion rules are improved by refining and decomposing the global conflict into local conflicts, which eliminates the differentiation in the body of evidence and improves the credibility of the fusion results. Comparative analysis results of the proposed method and other methods^9,12,17,18^ using field inspection data are shown in Table 11 and Fig. 9. The mass functions of levels I, II, and IV obtained in Ref.^9^ are close. Although the risk level can be identified and risk assessment can be performed normally, the mass functions are too close to each other. This closeness can easily cause risk assessment errors, and the fusion is susceptible to the influence of individual bodies of evidence and less resistant to interference. References^12,17^ can effectively identify the risk level, and the difference in mass function for each risk level is large. The resistance to interference is high, but m(Θ) is higher than 0.2. This result indicates that the uncertainty of risk indicators for the global risk is too high, and more risk source data are often needed to ensure an accurate assessment. The mass function obtained in Ref.^18^ is essentially similar among all risk levels, and m(Θ) is higher than 0.2. Hence, the uncertainty of the global risk is too high for a valid risk assessment. However, the gap between the mass functions of each risk level is significantly larger, and m(Θ) is less than 0.1 in this study. Thus, is has less impact on the global risk assessment as well as higher robustness and reliability.

**Table 11:** Results of different data fusion methods.

Fusion methods	Fusion results of site inspection indicators					Final fusion results					Risk level
	m(I)	m(II)	m(III)	m(IV)	m(Θ)	m(I)	m(II)	m(III)	m(IV)	m(Θ)	
Reference^9^	0.150	0.380	0.170	0.250	0.050	0.209	0.402	0.020	0.357	0.012	II
Reference^12^	0.000	0.600	0.100	0.000	0.300	0.010	0.580	0.100	0.008	0.302	II
Reference^17^	0.000	0.700	0.050	0.000	0.250	0.000	0.550	0.100	0.100	0.250	II
Reference^18^	0.240	0.310	0.120	0.150	0.180	0.287	0.262	0.112	0.137	0.202	I
Method in this paper	0.050	0.930	0.000	0.000	0.020	0.080	0.750	0.080	0.007	0.083	II

**Figure 9:**
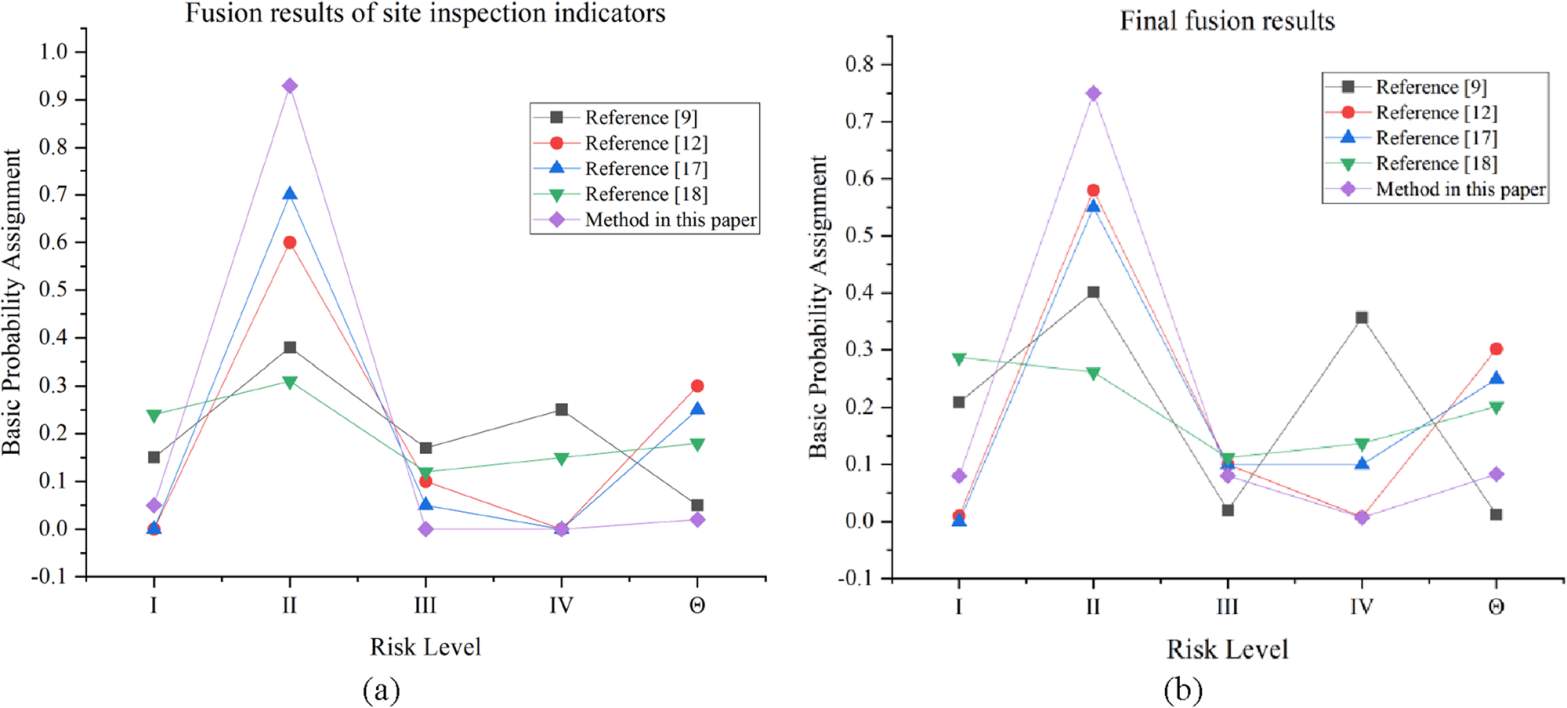
(**a**) Fusion results of site inspection indicators. (**b**) Final fusion results.
2. Three conflict indicators are considered to make the conflict feature extraction more comprehensive and improve the convergence speed of the fusion results. The above analysis shows that the proposed method obtains more realistic assessment results than those of other methods. The quality of a model is closely related to its convergence. BPA represents the certainty of the risk level. The faster BPA reaches stability, the faster the convergence speed of the model. Therefore, changes in the BPA can be used to analyze the convergence of the model. Here, the mass function is used as the objective function. When the BPA variation is less than the set threshold, the model can be considered to converge; the set threshold of this study is 0.05. The amount of data required for each method to reach performance stability is demonstrated below. The convergence of the model is only related to the BPA variation of the indicator, so the same results are obtained from using different risk sources to analyze the model convergence. Taking indicator F11 as an example, we see that the risk level of indicator F11 is level I. The BPA of level I becomes larger with the increase in the data fusion rounds, while the BPA of level II, level III, and level IV decreases. If the BPA variation is less than the threshold, the model can be considered to be convergent, whether the BPA increases or decreases. Twenty days of fusion results were obtained, as shown in Fig. 10. The BPAs of different methods are observed to maintain an upward trend before being stable. The BPA shows an upward trend before point A, but its fluctuation range remains within 0.05. Therefore, point A is the convergence point. Its abscissa indicates that the model convergence needs to be fused in four rounds. Points B, C, D, and E also represent convergence points. We can see that the proposed method requires minimal fusion rounds (four rounds). Its mass function oscillation can be maintained within 0.05, and its performance is relatively stable. However, in Ref.^9^, convergence begins after seven rounds of data fusion, whereas it begins after eight, ten, fourteen rounds for Refs.^12,17,18^, respectively. Therefore, the proposed method has good convergence.

**Figure 10:**
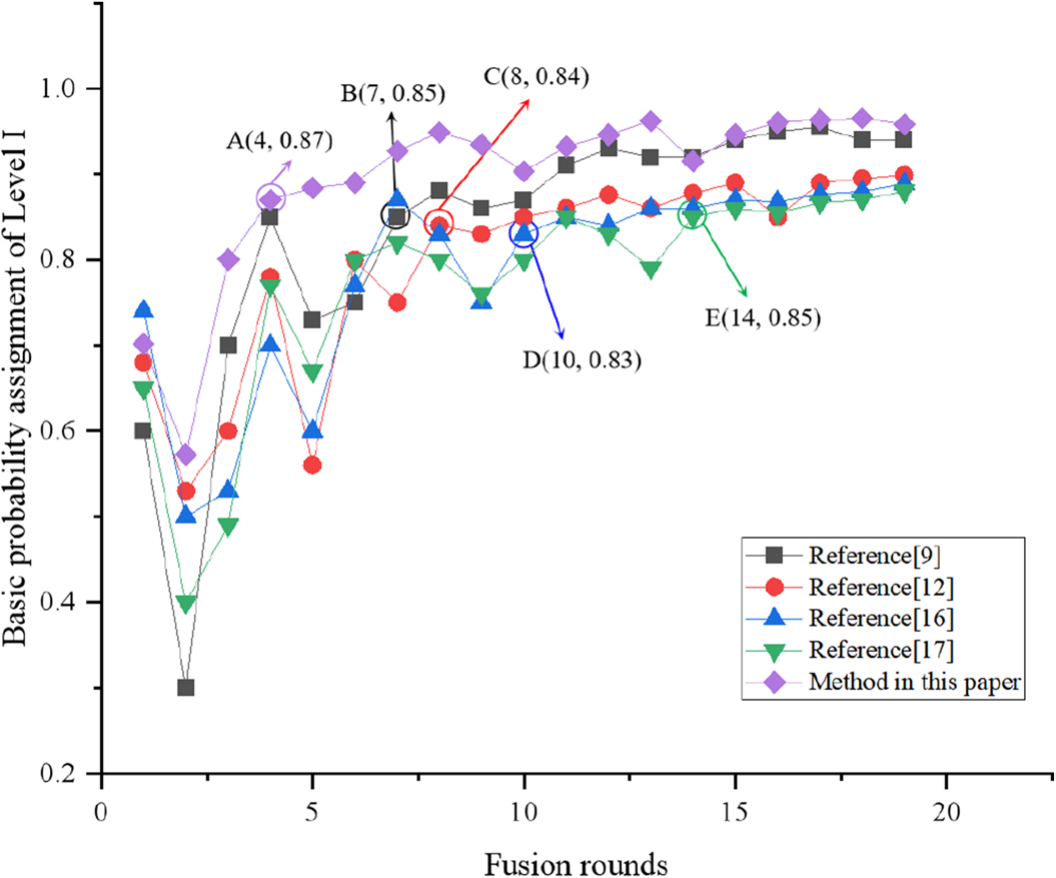
Comparison of mass function of level I. A(4, 0.87) is the convergence point of the proposed method. B(7, 0.85) is the convergence point of the method in Ref.^9^. C(8, 0.84) is the convergence point of the method in Ref.^12^. D(10, 0.83) is the convergence point of the method in Ref.^16^. E(14, 0.85) is the convergence point of the method in Ref.^17^.

The above analysis shows that the proposed method has high confidence and strong convergence compared to those of other methods. The global uncertainty of the proposed method is much lower, and the certainty of the assessment results is much higher, providing decision-makers with more accurate assessment results. The method can provide a timely warning of construction risks and prevent accidents. In summary, the proposed method can effectively reduce the incidence of construction accidents, improve the personal safety of workers, and promote the sustainable development of the construction industry.

## Conclusions

This study aimed to address the ambiguity and conflicting information problems of multi-source data fusion in subway pit collapse risk assessment. Hence, a method with strong convergence and high confidence based on the CM and improved D–S evidence theory is proposed. The method defines a new parameter by introducing conflict degree, discrepancy degree, and uncertainty. To improve fusion rules, the evidence focal element assignment is considered, and the risk level is obtained according to the maximum affiliation principle. Thus, a rapid and accurate assessment of the risk of pit collapse is realized, enabling construction workers to perceive the risk in time and providing decision-makers with more response time, which considerably reduces accidents. The proposed method was applied at Luochongwei Station of Guangzhou Metro Line 13. The following conclusions are obtained:

1. Combined with actual engineering cases, the four major indicators of survey and design, site inspection, instrument monitoring, and video surveillance are considered, and a risk assessment index system is constructed in many aspects to provide a research basis for the multi-data-source fusion risk assessment of subway foundation pit construction collapse.
2. When a single information source is used to assess the collapse risk, the results often contain deviations due to the uncertainty of the data. The proposed multi-source information fusion method comprehensively considers four types of risk source data, including survey and design, instrument monitoring, site inspection and video surveillance. The proposed method more comprehensively considers the construction site, which can reduce the data uncertainty and improve the risk assessment accuracy. Therefore, compared with the single-information-source risk assessment method, the multi-source information fusion assessment results often have a higher accuracy.
3. Conflicts between evidence bodies and conflicts caused by defects in fusion rules are considered simultaneously, which offers a high credibility and strong convergence. The risk assessment results obtained by the proposed method are such that the difference between the maximum value and the second largest value of the BPA is greater than 0.2. Moreover, the global uncertainty is less than 0.1. However, other methods can only satisfy one or neither, indicating that the proposed method has a high credibility. The convergence of the proposed model is only related to the variation in BPA, and similar results are obtained from using different risk source data to evaluate the model convergence. By analyzing the multi-period data of a single indicator (instrument monitoring), we find that the other existing methods need at least seven cycles of data fusion before convergence begins, whereas the proposed method reaches convergence in four cycles, indicating that the proposed method converges quickly.

Nevertheless, the proposed method also has some limitations. First, because the amount of data is relatively small, it is necessary to develop a set of risk assessment data acquisition systems for coastal cities such as Guangdong. In addition, the proposed method cannot predict the risk status of the next construction process, which necessitates further research.

## Data Availability

The datasets generated and analyzed during the current study are not publicly available but are available from the corresponding author on reasonable request.
